# Traditional Dairy and Olive Food Preferences Are Associated with Mediterranean Diet Adherence in Cyprus

**DOI:** 10.3390/foods15173024

**Published:** 2026-08-27

**Authors:** Eleni P. Andreou, Panayiota Tsokkou, Panayiota Theophilou, Christina Kritikou, Abdallah Kanaan, Stella A. Nicolaou, Evelina Charidemou, Yiannis Sarigiannis, Christoforos D. Giannaki, Christoforos Christoforou, Christos Papaneophytou

**Affiliations:** 1 Department of Life Sciences, School of Life and Health Sciences, University of Nicosia, 2417 Nicosia, Cyprus; tsokkou.p@live.unic.ac.cy (P.T.); p_theophilou79@hotmail.com (P.T.); kriticouchris@gmail.com (C.K.); aakanan18@agr.just.edu.jo (A.K.); nicolaou.s@unic.ac.cy (S.A.N.); charidemou.e@unic.ac.cy (E.C.); giannaki.c@unic.ac.cy (C.D.G.); christoforou.c@unic.ac.cy (C.C.); 2 Research Centre for Exercise and Nutrition (RECEN), University of Nicosia, 2417 Nicosia, Cyprus; 3 Department of Health Sciences, School of Life and Health Sciences, University of Nicosia, UNIC Athens, 167 77 Athens, Greece; sarigiannis.i@unic.ac.cy

**Keywords:** mediterranean diet, MEDAS, olive oil, dairy products, food preferences, olive products, Cyprus, dietary adherence, nutrition, cross-sectional study

## Abstract

The Mediterranean diet is recognised as one of the healthiest dietary patterns worldwide; however, adherence has declined even within Mediterranean populations. This study investigated Mediterranean diet adherence and examined its associations with preferences for traditional dairy products and olive-related products among adults participating in a study in Cyprus. A cross-sectional online survey was conducted among 273 adults using a structured questionnaire that included sociodemographic and lifestyle characteristics, the 14-item Mediterranean Diet Adherence Screener (MEDAS), and questions assessing preferences for and consumption of dairy products, olive oil, olive-derived products, and foods prepared with olive oil. Descriptive statistics, principal component analysis, and multiple linear regression were performed. The mean MEDAS score was 8.1 ± 2.6; using study-specific descriptive categories, 40.0% of participants had scores classified as good to excellent adherence. Olive oil was the most preferred and most frequently consumed olive-related product, with 75.1% of participants reporting that they “liked very much” olive oil and 49.8% consuming it daily. In the primary multivariable analysis, older age, greater traditional dairy preference, greater olive-related preference, and more frequent olive-related consumption were associated with higher MEDAS scores, whereas sex, body mass index, smoking status, and physical activity were not significantly associated with MEDAS score. However, in a sensitivity analysis excluding the two olive-oil-related MEDAS components, the olive-related preference and consumption associations were no longer statistically significant. Overall, traditional food preferences were associated with Mediterranean diet adherence within this convenience sample; however, the olive-related findings should be interpreted cautiously because of potential construct and measurement overlap. Given the cross-sectional design and demographic characteristics of the sample, these findings do not establish temporality or causality and should not be considered representative of the general adult population of Cyprus.

## 1. Introduction

The Mediterranean diet (MD) is widely recognised as one of the healthiest dietary patterns worldwide and has been consistently associated with improved health outcomes and a reduced risk of chronic diseases [1,2,3]. Characterised by a high intake of fruits, vegetables, legumes, whole grains, nuts, and olive oil as the principal source of dietary fat, together with moderate consumption of fish and dairy products and limited intake of red and processed meat, the Mediterranean diet represents both a nutritionally balanced dietary model and a cultural tradition shared among Mediterranean countries. Owing to its nutritional, cultural and environmental significance, it has been recognised by UNESCO as an Intangible Cultural Heritage of Humanity [4]. Evidence from prospective cohort studies and clinical trials has demonstrated that greater adherence to the Mediterranean diet is associated with lower risks of cardiovascular disease, obesity, type 2 diabetes mellitus, certain cancers, cognitive decline and all-cause mortality [2,3]. Emerging evidence suggests that adherence to the Mediterranean diet is associated with favourable modulation of the gut microbiota and inflammatory processes, which may contribute to its broader health effects [5]. Despite its Mediterranean heritage, relatively few studies have investigated adherence to the Mediterranean diet in Cyprus. Recent evidence from Greek and Cypriot adults showed that higher adherence to the Mediterranean diet was associated with greater subjective well-being, highlighting the importance of identifying the factors that promote adherence within the Cypriot population [6].

Dairy products provide high-quality protein, calcium, vitamin B12, riboflavin, and other essential nutrients that contribute to nutritional adequacy and support musculoskeletal health [7,8]. Traditional Mediterranean dietary patterns generally include moderate consumption of dairy products, particularly fermented products such as yogurt and cheese. Fermented dairy products may possess distinct nutritional and functional properties resulting from microbial activity and the formation of bioactive compounds during fermentation [9]. However, consumer attitudes toward dairy products have evolved in recent years in response to health, environmental, and market-related considerations. Because food preferences are important influences on food choice and habitual dietary intake, understanding contemporary preferences for milk and dairy products may provide useful insight into dietary behaviour and is relevant to nutrition research and public health [7,10].

Cyprus represents a distinctive Mediterranean setting in which traditional dairy production remains an important component of both the national economy and cultural identity. Halloumi cheese, recognised as a Protected Designation of Origin (PDO) product, together with other traditional dairy products such as Anari, yoghurt and fermented milk beverages, continues to play a central role in the Cypriot diet. Nevertheless, increasing Westernisation and changing dietary behaviours may influence the consumption of these traditional foods, potentially reducing adherence to the Mediterranean dietary pattern.

Olive oil is the hallmark of the Mediterranean diet and one of the most extensively studied functional foods because of its unique nutritional composition. Extra virgin olive oil is rich in monounsaturated fatty acids, particularly oleic acid, together with bioactive compounds including polyphenols, tocopherols and phytosterols that exhibit antioxidant and anti-inflammatory properties [2,11]. Evidence from major intervention studies, including the PREDIMED trial, has demonstrated that regular consumption of extra virgin olive oil contributes to improved cardiovascular health, favourable lipid metabolism and a lower incidence of chronic disease [2]. In addition, emerging evidence suggests that olive oil may exert neuroprotective effects through reductions in oxidative stress, neuroinflammation and vascular dysfunction. A recent systematic review by Kanaan et al. [12] concluded that the fatty acid profile and bioactive compounds of olive oil may positively influence cognitive function and healthy brain ageing, although additional high-quality clinical studies are required to strengthen the evidence.

Beyond olive oil itself, table olives and other olive-derived products contribute substantially to dietary diversity within Mediterranean populations and remain culturally, nutritionally and economically important in Cyprus. Consumer preference for these traditional foods may influence habitual dietary intake and, consequently, overall adherence to the Mediterranean diet. However, relatively little is known about preferences for different olive-derived products or the extent to which these preferences are associated with adherence to the Mediterranean diet.

Mediterranean countries have undergone a nutrition transition characterised by declining adherence to the traditional Mediterranean diet and increasing consumption of ultra-processed and convenience foods, particularly among younger populations, reflecting the progressive Westernisation of dietary habits [13]. Similar trends have been reported in Cyprus and may contribute to declining adherence to traditional Mediterranean dietary practices. Because food choice is influenced by biological, psychological, cultural, social and economic factors, including taste preferences, perceived health benefits, affordability, convenience, sustainability, food availability and cultural traditions [14,15], understanding the determinants of food preferences is essential for developing effective nutrition education programmes and culturally appropriate public health interventions. As food preferences are associated with habitual dietary intake, examining these preferences may provide insight into patterns of food choice and overall dietary quality. Adherence to the Mediterranean diet is commonly assessed using validated dietary indices such as the Mediterranean Diet Adherence Screener (MEDAS), which has been widely applied in epidemiological and intervention studies to evaluate dietary quality and predict health outcomes [2]. In addition to its recognised protective effects against cardiovascular disease, type 2 diabetes and other chronic conditions, emerging evidence indicates that greater adherence to the Mediterranean diet following a cancer diagnosis is associated with improved survivorship outcomes, including reduced cancer recurrence risk and lower cancer-related fatigue, particularly among colorectal cancer survivors [16].

Food preferences represent an important influence on food choice and habitual dietary intake and are shaped by multiple sensory, cultural, behavioural and environmental factors [6,14]. Within Mediterranean populations, preferences for culturally familiar foods may therefore be associated with the extent to which traditional dietary practices are followed. This relationship should not necessarily be regarded as unidirectional, however, as food preferences may be associated with food selection and consumption, while repeated exposure to foods through habitual dietary practices may also shape preferences.

Despite the well-established health benefits of the Mediterranean dietary pattern, limited evidence is available regarding the relationship between consumer preferences for traditional Mediterranean foods, particularly traditional dairy products, olive oil and olive-derived products, and overall Mediterranean diet adherence among Mediterranean populations. Although previous studies [6,14] have examined sociodemographic, lifestyle and behavioural factors associated with Mediterranean diet adherence, the relationship between preferences for culturally relevant traditional foods and adherence to the overall dietary pattern remains comparatively underexplored. To our knowledge, no previous study has comprehensively examined the relationships between Mediterranean diet adherence and preferences for and consumption patterns of traditional dairy products, olive oil and olive-derived products among adults living in Cyprus. Addressing this knowledge gap may contribute to a better understanding of the behavioural and cultural correlates of Mediterranean dietary practices and may inform future research on culturally appropriate approaches to preserving traditional food practices, supporting local agricultural production and informing evidence-based nutrition policies and interventions.

Therefore, the present study aimed to investigate preferences for and consumption patterns of milk and dairy products, olive-related items (olive oil and olive-derived products) among adults participating in this study in Cyprus and to examine their associations with Mediterranean diet adherence. In addition, the study examined whether traditional dairy preference and olive oil and olive-derived product preference and consumption were associated with Mediterranean diet adherence after accounting for selected demographic, anthropometric and lifestyle characteristics. Given the cross-sectional design, the study was intended to identify associations rather than establish temporal or causal relationships.

## 2. Methods

### 2.1. Study Design

A cross-sectional observational study was conducted among adults living in Cyprus to investigate dietary preferences and consumption habits relating to milk, dairy products, olive oil and olive-derived products and their association with adherence to the Mediterranean diet. Data were collected using a structured online questionnaire between June 2024 and May 2025.

### 2.2. Study Population and Participant Recruitment

Adults aged ≥ 18 years who were permanent residents of Cyprus were eligible to participate. Individuals younger than 18 years or those who did not provide informed consent were excluded.

Participants were recruited using convenience sampling through social media platforms, university mailing lists, professional networks and community organisations. Recruitment was conducted online to facilitate participation from adults across different regions of Cyprus. As participation was voluntary, the study sample was not intended to be nationally representative. Before completing the questionnaire, participants received an electronic information sheet describing the study objectives, confidentiality procedures and the voluntary nature of participation. Electronic informed consent was obtained before questionnaire completion.

A total of 273 participants completed the questionnaire and were included in the final analysis. This sample size exceeded commonly recommended minimum requirements for exploratory factor analysis and provided an adequate number of observations relative to the predictors included in the multiple linear regression model, supporting stable and reliable parameter estimation [17].

### 2.3. Questionnaire Development

Data were collected using a structured, self-administered questionnaire developed in Google Forms and informed by a review of the literature on dietary preferences, food-choice behaviour, and Mediterranean diet adherence. The questionnaire combined a validated dietary assessment instrument with study-specific items assessing preferences for and consumption of dairy products and olive-related items.

The questionnaire was administered in Greek. The study-specific food-preference and consumption items were originally developed in Greek and therefore did not require translation or back-translation. Mediterranean diet adherence was assessed using the validated Greek-language version of the 14-item Mediterranean Diet Adherence Screener (MEDAS), originally developed within the framework of the PREDIMED study [2] and subsequently developed and validated for use in Greek by Michalis and Costarelli [18]. Accordingly, no additional translation or back-translation of MEDAS was undertaken for the present study. Mediterranean diet adherence was assessed using the validated Greek-language version of the 14-item Mediterranean Diet Adherence Screener (MEDAS) [2,18]. Each item contributes 0 or 1 point, yielding a total score ranging from 0 to 14, with higher scores indicating greater adherence to the Mediterranean diet. For descriptive presentation in the present study, total MEDAS scores were grouped into five study-specific categories: low (≤5), moderate (6–7), good (8), very good (9), and excellent (≥10). These categories were used solely to facilitate descriptive interpretation of the score distribution and do not represent externally validated MEDAS adherence thresholds. The continuous MEDAS score was used in the multivariable regression analyses.

The dairy-product item pool comprised 61 response fields and was developed following a review of the relevant literature on dairy consumption and food preferences and consideration of milk and dairy products commonly available and consumed in Cyprus. To ensure that the item pool reflected the local food environment and the range of products available to consumers, product selection was additionally informed by the CyprusAtlas dairy-products directory and relevant information from the Ministry of Energy, Commerce and Industry of the Republic of Cyprus. The resulting item pool included traditional Cypriot dairy products as well as other commercially available products commonly accessible to consumers in Cyprus, including milk, yogurt, cheese, fermented dairy products, and specialty dairy products. The 61st response field was an open “Other” option. As no responses were recorded for this field, it was excluded from the component analysis; consequently, 60 substantive dairy-preference items were included in the principal component analysis (PCA).

The six olive-related items were selected to represent commonly consumed forms and culinary uses of olives and olive oil relevant to the Cypriot dietary context. For clarity and consistency throughout the manuscript, the term “olive-related items” is used as an umbrella term encompassing three categories assessed in the questionnaire: (i) olive oil; (ii) olive-derived products, comprising green olives, black olives, and olive paste; and (iii) culinary uses of olive oil, comprising olive oil used as a bread spread and foods prepared with olive oil. Where appropriate, the specific category or individual item is stated explicitly. The items were selected based on the relevant literature and consideration of customary Mediterranean and Cypriot dietary practices, with the aim of capturing preferences across olive oil, olive-derived foods, and common culinary uses of olive oil.

Food preferences for dairy products and olive-related items were assessed using five-point hedonic (Likert-type) scales ranging from 1 (strongly dislike) to 5 (like very much). Consumption frequency was assessed using predefined categorical response options. Composite preference and consumption variables were derived from the corresponding questionnaire items for use in the multivariable analyses, as described in Section Composite Score Calculation.

Prior to pilot testing, the complete questionnaire was reviewed by a panel of five experts with relevant expertise in nutrition, dietetics, food science, and Mediterranean dietary practices. The expert panel evaluated the questionnaire as a whole, including the study-specific food-preference and consumption items, with respect to content relevance, clarity, comprehensibility, appropriateness of the response options, and suitability for the study objectives and the Cypriot dietary context. Feedback from the expert panel was used to refine the questionnaire before pilot testing.

The questionnaire was subsequently pilot-tested among the first 20 participants to assess the clarity and comprehensibility of the questions, appropriateness of the response options, and ease of completion. Based on feedback obtained during pilot testing, minor wording changes were made to improve clarity without altering the content, meaning, response scales, or scoring of the questionnaire items. Because these modifications were limited to wording and did not affect the substantive content of the questionnaire, data from the 20 pilot participants were retained in the final analytical sample.

A more comprehensive evaluation of the psychometric properties of the study-specific questionnaire components is being undertaken separately; therefore, the present study reports the psychometric analyses directly relevant to the current sample. Internal consistency was assessed using Cronbach’s alpha for the dairy preference questionnaire and the 6-item olive-related preference questionnaire. The dimensional structure of the substantive dairy-preference items was examined using PCA, as described in Section 2.7.

The questionnaire comprised the following sections:

- sociodemographic characteristics (age, sex, education, marital status, occupation, monthly income, and area of residence);
- anthropometric characteristics (self-reported body weight and height);
- lifestyle behaviours, including smoking status, physical activity, and general health status;
- dietary habits and meal frequency;
- Mediterranean Diet Adherence Screener (MEDAS);
- consumption habits and preferences for milk and dairy products; and
- consumption habits and preferences for olive-related items.

The present analyses focused on Mediterranean diet adherence, assessed using MEDAS, together with preferences for and consumption of traditional dairy products and olive-related items. Sociodemographic, anthropometric, and lifestyle variables were included as descriptive characteristics and, where appropriate, as covariates in the multivariable analyses.

#### Composite Score Calculation

Three composite variables were derived for use in the multivariable analyses: the traditional dairy preference score, the olive-related preference score, and the olive-related consumption-frequency score.

The traditional dairy preference score was calculated as the mean of the preference ratings for the dairy products assigned to the traditional dairy preference component identified by PCA. Each contributing item was assessed using a five-point hedonic scale ranging from 1 (strongly dislike) to 5 (like very much), with “Not applicable” responses treated as missing. The resulting composite retained a possible score range of 1–5, with higher scores indicating greater overall preference for traditional dairy products.

The olive-related preference score was calculated as the mean of the six preference items assessing olive oil, green olives, black olives, olive paste, olive oil used as a bread spread, and foods prepared with olive oil. Each item was scored from 1 (strongly dislike) to 5 (like very much), with “Not applicable” responses treated as missing. The resulting composite score had a possible range of 1–5, with higher scores indicating greater overall preference for olive-related items.

The olive-related consumption-frequency score was calculated as the mean of the corresponding six consumption-frequency items. Responses were coded as 1 = never, 2 = rarely, 3 = 1–2 times/week, 4 = 3–4 times/week, and 5 = daily. The resulting composite score had a possible range of 1–5, with higher scores indicating more frequent overall consumption of olive-related items.

The traditional dairy preference score, olive-related preference score, and olive-related consumption-frequency score were treated as continuous variables and entered as independent variables in the multiple linear regression analysis examining factors associated with Mediterranean diet adherence, as measured by the MEDAS score.

### 2.4. Assessment of Mediterranean Diet Adherence

Adherence to the Mediterranean dietary pattern was assessed using the validated 14-item MEDAS, originally developed for the PREDIMED study. Each item was scored according to the established MEDAS criteria, yielding a total score ranging from 0 to 14, with higher scores indicating greater adherence to the Mediterranean diet.

The MEDAS assesses habitual consumption of key components of the Mediterranean diet, including olive oil, vegetables, fruits, legumes, fish, nuts, wine, red and processed meat, sugar-sweetened beverages and commercial sweets.

### 2.5. Assessment of Food Preferences

Participants were asked to evaluate their preferences and consumption frequency for a wide range of milk and dairy products and olive-related items commonly consumed in Cyprus and the broader Mediterranean region. Halloumi and Anari were included as traditional Cypriot dairy products.

The dairy section included fresh milk, lactose-free milk, plant-based milk alternatives, yoghurt, Halloumi cheese, Anari cheese and other traditional and commercial dairy products.

The olive section included olive oil, green olives, black olives, olive paste, olive oil bread spread and foods prepared with olive oil. Although foods prepared with olive oil are not olive-derived products, they were included to capture preferences for olive oil-based culinary practices that characterise the Mediterranean dietary pattern. Throughout the manuscript, the term olive-related items refers collectively to olive oil, olive-derived products and foods prepared with olive oil, unless otherwise specified.

Preference scores were calculated by averaging responses within each food category, with higher scores indicating stronger consumer preference. Food preferences were assessed using a five-point hedonic scale ranging from 1 (strongly dislike) to 5 (like very much).

### 2.6. Ethical Considerations

The study was conducted in accordance with the ethical principles of the Declaration of Helsinki. It formed part of an ongoing research project approved by the Cyprus National Bioethics Committee (Approval No. EEBKΕΠ2020.01.66). All participants received an electronic participant information sheet and provided electronic informed consent before completing the questionnaire. Participation was voluntary and anonymous, and no personally identifiable information was collected. Participants could withdraw from the study at any time before submitting their responses.

### 2.7. Statistical Analysis

Statistical analyses were performed using IBM SPSS Statistics for Windows, Version 29.0 (IBM Corp., Armonk, NY, USA). Continuous variables are presented as means ± standard deviations (SD), and categorical variables as frequencies and percentages. For descriptive analyses, age was categorised into four groups (18–29, 30–44, 45–59, and ≥60 years), whereas age was entered as a continuous variable in the multivariable regression analyses.

All questionnaire items required for the present analyses were mandatory in the electronic form and therefore could not be left unanswered. However, “Not applicable” was available as a response option for food-preference items. Because this response did not represent a preference rating on the 1–5 hedonic scale, it was not assigned a numerical value and was treated as missing for statistical analysis. For participant-level composite preference scores, “Not applicable” responses were excluded and scores were calculated from the available valid item responses according to the procedures described in Section 2.3. For PCA, cases with missing values among the included dairy-preference items were excluded listwise. No missing values were imputed.

Internal consistency was assessed using Cronbach’s alpha for the dairy-preference questionnaire and the six-item olive-related preference scale. The dairy-preference questionnaire comprised 61 response fields, including an “Other” option. Because no responses were recorded for the “Other” field, it was excluded from the component analysis, leaving 60 substantive dairy-preference items for PCA.

The dimensional structure of the dairy-preference items was explored using principal component analysis (PCA). PCA was selected as an exploratory data-reduction approach to identify groups of correlated preference items and derive interpretable preference dimensions. Sampling adequacy and the suitability of the correlation matrix for PCA were assessed using the Kaiser–Meyer–Olkin (KMO) measure and Bartlett’s test of sphericity, respectively. Varimax orthogonal rotation was applied to facilitate interpretation by maximising differentiation among component loadings. Components were retained based on eigenvalues >1.0 together with the interpretability of the resulting component structure. Component labels were assigned according to the items with the strongest rotated loadings. The complete rotated component loading matrix is provided in Supplementary Table S2.

Composite scores for traditional dairy preference, olive-related preference, and olive-related consumption frequency were calculated according to the scoring procedures described in Section 2.3. Higher scores indicated greater preference or, for the consumption-frequency score, more frequent consumption. Composite scores were treated as continuous variables in the multivariable regression analyses.

Differences in individual Mediterranean Diet Adherence Screener (MEDAS) components between men and women were assessed using Pearson’s chi-square test.

Multiple linear regression analysis was performed to examine factors associated with Mediterranean diet adherence, with the total MEDAS score entered as the dependent variable. Age, sex, body mass index (BMI), smoking status, physical activity, traditional dairy preference score, olive-related preference score, and olive-related consumption-frequency score were entered simultaneously as independent variables. Covariates were selected according to their relevance to the study objectives and included demographic, anthropometric, lifestyle, and food-related characteristics. Socioeconomic variables, including education and household income, were collected for descriptive characterisation but were not included in the primary regression model; therefore, residual confounding by socioeconomic position cannot be excluded. Regression results are reported as unstandardised regression coefficients (B), standard errors (SE), standardised regression coefficients (β), t-values, and *p*-values. Overall model fit is reported using R^2^, adjusted R^2^, the standard error of the estimate, and the F-statistic.

Prior to interpretation of the regression model, diagnostic procedures were performed to assess relevant assumptions of multiple linear regression. Linearity and homoscedasticity were evaluated by visual inspection of plots of standardised residuals against standardised predicted values. Approximate normality of the residuals was assessed by inspection of the distribution of standardised residuals and the normal probability (P–P) plot.

To assess the potential influence of construct and measurement overlap between the olive-related independent variables and MEDAS, a sensitivity analysis was performed using a modified MEDAS score excluding the two olive-oil-related items (use of olive oil as the principal culinary fat and consumption of ≥4 tablespoons of olive oil per day), resulting in a theoretical score range of 0–12. The multiple linear regression analysis was repeated using the modified MEDAS score as the dependent variable and the same independent variables as in the primary model. Regression analyses were based on complete cases for all variables included in each model; the sensitivity analysis included 265 participants. Complete results of this analysis are presented in Supplementary Table S3.

All statistical tests were two-sided, and a *p*-value < 0.05 was considered statistically significant.

### 2.8. Data Availability

The datasets generated and analysed during the current study are available from the corresponding author upon reasonable request. Data are not publicly available because they contain information that could compromise participant privacy and confidentiality.

## 3. Results

### 3.1. Participant Characteristics

A total of 273 adults were included in the final analysis (Table 1). The sociodemographic, anthropometric, and lifestyle characteristics of the participants are presented in Table 1. The mean age was 35.3 ± 11.6 years. Approximately half of the participants (49.8%) were aged 18–29 years, followed by 29.7% aged 30–44 years, 16.8% aged 45–59 years, and 3.7% aged ≥ 60 years. Females comprised 70.7% of the study sample.

Most participants had completed tertiary education, with 48.7% holding an undergraduate degree and 37.7% a postgraduate degree, while 13.6% had completed secondary education. Based on BMI classification, 50.9% of participants had a normal body weight, 32.6% were overweight, 10.6% were obese, and 5.9% were underweight.

Most participants were non-smokers (77.7%), whereas 22.3% reported current smoking. Regarding occupational activity, almost half of the participants (47.6%) reported predominantly sedentary occupations, followed by occupations involving walking for several hours per day (33.7%). Smaller proportions reported occupations involving walking with physical effort (11.0%), heavy manual work (1.8%) or other activity patterns (5.9%).

### 3.2. Mediterranean Diet Adherence

Mediterranean diet adherence was assessed using the 14-item Mediterranean Diet Adherence Screener (MEDAS). Complete MEDAS data were available for 273 participants. The mean MEDAS score was 8.1 ± 2.6, with a median score of 8 and scores ranging from 3 to 14 (Table 2).

Based on the predefined MEDAS categories (Table 3), approximately one-third of participants demonstrated low adherence (29.7%), while 30.4% exhibited moderate adherence. A further 15.8% achieved a score of 8, indicating good adherence, whereas 10.3% and 13.9% were classified as having very good and excellent adherence, respectively. Overall, 40.0% of participants achieved a MEDAS score of ≥8, reflecting good to excellent adherence to the Mediterranean dietary pattern.

#### Individual Adherence to the Mediterranean Diet (MEDAS Items)

Table 4 presents participants’ responses to the 14 individual items of the MEDAS. Overall, the highest adherence was observed for the use of olive oil as the principal culinary fat, with 245 participants (89.7%) meeting this criterion. Approximately one-third of participants reported consuming sofrito-based dishes at least twice per week (33.3%) and preferring poultry or rabbit over red or processed meat (34.8%). Adherence to limiting commercial sweets to fewer than three servings per week (27.5%), consuming nuts at least once weekly (27.1%), and limiting butter, margarine, or cream intake to less than one serving per day (26.0%) was moderate.

Lower adherence was observed for several Mediterranean dietary recommendations. Only 24.5% of participants consumed at least two servings of vegetables daily, while 17.6% consumed at least three servings of fruit per day. Similarly, 21.6% reported consuming legumes at least three times per week, 14.3% met the recommendation for fish or seafood intake, and only 17.9% consumed four or more tablespoons of olive oil per day. Compliance with the recommendation of consuming three or more glasses of wine per week was the lowest, with only 7.7% of participants meeting this criterion.

When stratified by sex, men generally demonstrated slightly higher adherence than women for vegetable intake (29.1% vs. 22.7%), fish and seafood consumption (19.0% vs. 12.4%), legumes (26.6% vs. 19.6%), butter/margarine restriction (31.6% vs. 23.7%), and sofrito consumption (39.2% vs. 30.9%). Conversely, women showed marginally higher adherence to the use of olive oil as the main culinary fat (90.7% vs. 87.3%). However, none of the individual MEDAS items differed significantly between men and women (all Pearson’s chi-square *p* > 0.05).

### 3.3. Consumer Preferences for Milk and Dairy Products

**Figure 1:**
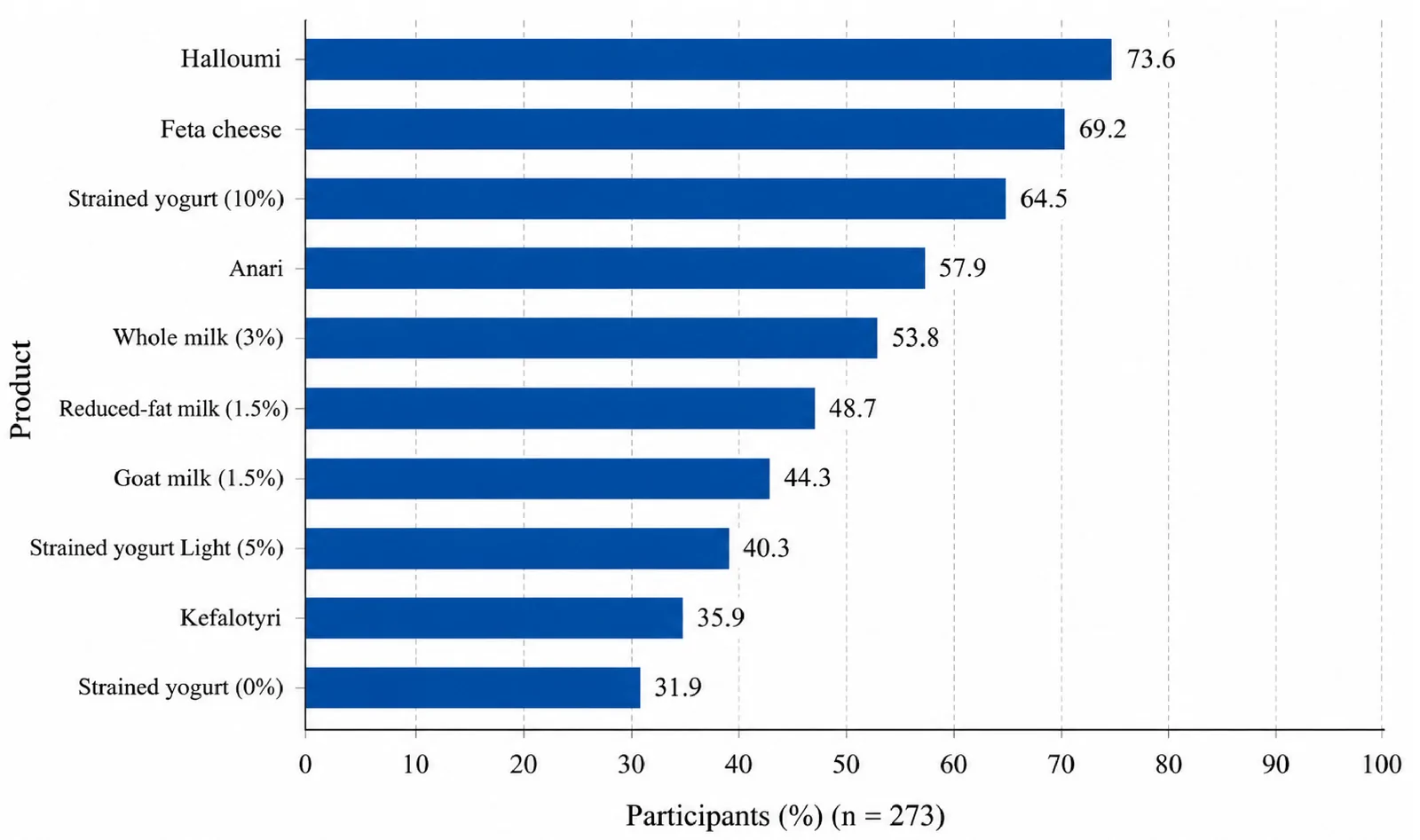
Percentage of participants selecting “Like very much” for the ten most preferred milk and dairy products among the study participants (n = 273).

Participants evaluated 60 milk and dairy products using a five-point hedonic scale ranging from 1 (strongly dislike) to 5 (like very much). The ten highest-ranked products, based on the proportion of participants selecting the response “Like very much”, are presented in Table 5, while Figure 1 illustrates the corresponding distribution.

Traditional dairy products received the highest preference ratings. Halloumi was the most preferred product, with 201 participants (73.6%) indicating that they liked it very much, followed by feta cheese (189 participants, 69.2%), strained yogurt (10%) (176 participants, 64.5%), and Anari (158 participants, 57.9%). Among milk products, whole milk (3%) and reduced-fat milk (1.5%) also received high preference ratings, with 147 (53.8%) and 133 (48.7%) participants, respectively, selecting the highest hedonic category. Goat milk (1.5%), strained yogurt Light (5%), Kefalotyri, and strained yogurt (0%) completed the ten most preferred dairy products. As shown in Figure 1, traditional dairy products consistently received the highest preference ratings, whereas milk products showed comparatively lower, but still favourable, levels of consumer acceptance. The traditional dairy products can be of Cypriot or Greek origin or generally from the Mediterranean region. Descriptions of these traditional dairy products are provided in Supplementary Table S1.

Overall, participants demonstrated a clear preference for traditional dairy products, particularly local cheeses and fermented dairy products, indicating that these foods remain central components of dietary preferences among Cypriot consumers.

### 3.4. Reliability and Component Structure of the Dairy Preference Questionnaire

The internal consistency of the dairy preference questionnaire was assessed using Cronbach’s alpha. The questionnaire demonstrated excellent internal consistency (Cronbach’s α = 0.91), indicating a high degree of consistency among the dairy preference items.

The suitability of the data for principal component analysis (PCA) was supported by a Kaiser–Meyer–Olkin (KMO) measure of sampling adequacy of 0.88 and a significant Bartlett’s test of sphericity (*p* < 0.001). PCA with varimax rotation identified four interpretable components, which together accounted for 67.4% of the total variance. Following rotation, Component 1 (traditional cheeses) accounted for 25.42% of the variance, Component 2 (milk products) for 18.63%, Component 3 (yogurt products) for 12.27%, and Component 4 (specialty dairy products) for 11.08%. Component labels were assigned according to the dairy products with the strongest rotated component loadings. The variance accounted for by each component is presented in Table 6, and the complete rotated component loading matrix for the 60 substantive dairy-preference items included in the PCA is provided in Supplementary Table S2. The open “Other” response field contained no responses and was therefore excluded from the PCA.

Among participants with sufficient available data for calculation of the composite score (n = 269), the mean traditional dairy preference score was 3.93 ± 0.96 on a possible scale of 1–5.

### 3.5. Consumer Preferences for Olive-Related Items

**Figure 2:**
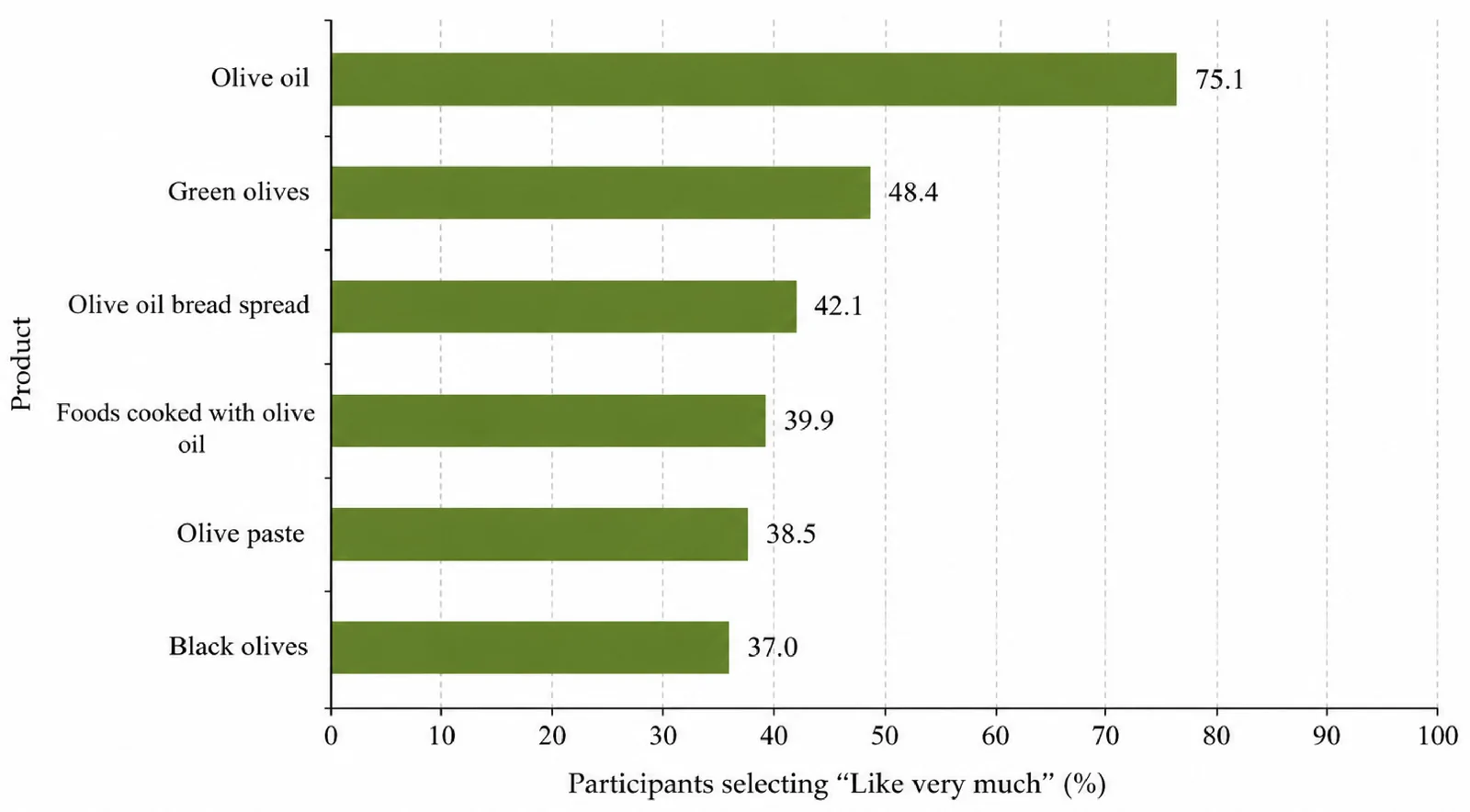
Most preferred olive-related items among the study participants (n = 273). Participants evaluated milk and dairy products using a five-point hedonic scale. The figure presents the number and percentage of participants who selected the highest response category, “Like very much.” Products are ranked in descending order according to this percentage.

The 6-item olive-related items preference questionnaire demonstrated very good internal consistency (Cronbach’s α = 0.89). Participants evaluated six olive-related items using a five-point hedonic scale ranging from 1 (strongly dislike) to 5 (like very much). The most preferred products are presented in Table 7, while the distribution of participants selecting the highest preference category is illustrated in Figure 2.

Olive oil was the most preferred product, with 205 participants (75.1%) reporting that they liked it very much. Green olives ranked second (132 participants, 48.4%), followed by olive oil bread spread (115 participants, 42.1%), foods cooked with olive oil (109 participants, 39.9%), olive paste (105 participants, 38.5%), and black olives (101 participants, 37.0%). Overall, olive oil received substantially higher preference ratings than the remaining olive-derived products, indicating its prominent role in the dietary habits of the study population. The mean overall olive-related preference composite score was 3.76 ± 1.15 on a possible scale of 1–5, indicating a generally favourable preference for olive-related items among the study participants.

Figure 2 presents the percentage of participants selecting “Like very much” for olive-related items among the study participants (N = 273). Olive oil received the highest preference rating (75.1%), followed by green olives and olive oil bread spread, demonstrating strong consumer preference for olive-based products within the study population.

### 3.6. Consumption Frequency of Olive-Related Items

The 6-item olive-related items consumption frequency composite demonstrated acceptable internal consistency (Cronbach’s α = 0.72). The frequency of consumption of olive products are presented in Table 8. Olive oil was the most frequently consumed product, with 136 participants (49.8%) reporting daily consumption and a further 83 participants (30.4%) consuming it 3–4 times per week. In contrast, daily consumption of green olives and black olives was uncommon, reported by only 3 participants (1.1%) for each product.

Green olives were most commonly consumed rarely (46.9%), followed by 1–2 times per week (23.4%), whereas black olives showed a similar consumption pattern, with 41.8% of participants reporting rare consumption and 23.8% consuming them 1–2 times per week. Olive paste and olive oil bread spread were consumed infrequently, with more than half of participants reporting rare or never consuming these products. Foods cooked with olive oil demonstrated a more balanced distribution, with approximately one-third of participants consuming them rarely (33.7%), while 12.1% reported daily consumption.

Overall, the findings indicate that olive oil is a staple component of the participants’ diet, whereas other olive-derived products are generally consumed less frequently. Across the six olive-related items, the mean overall consumption-frequency composite score was 2.53 ± 0.62 on a possible scale of 1–5, with higher scores representing more frequent consumption.

### 3.7. Multiple Linear Regression Analysis of Factors Associated with Mediterranean Diet Adherence

To identify factors independently associated with Mediterranean diet adherence, multiple linear regression analysis was performed using the MEDAS score as the dependent variable. Age, sex, body mass index (BMI), smoking status, physical activity, traditional dairy preference score, olive -related items preference score, and olive-related items consumption score were entered simultaneously as independent variables. Visual inspection of the regression diagnostic plots did not indicate substantial departures from linearity, homoscedasticity, or approximate normality of the residuals. The results of the regression analysis are presented in Table 9.

The overall regression model was statistically significant (F = 13.48, *p* < 0.001) and explained 29% of the variance in MEDAS score (R^2^ = 0.29; adjusted R^2^ = 0.27). Greater preference for traditional dairy products was independently associated with higher Mediterranean diet adherence (B = 0.51; β = 0.20; *p* = 0.008). Older age was also independently associated with higher MEDAS scores (B = 0.03; β = 0.15; *p* = 0.035).

Greater preference for traditional dairy products was independently associated with higher Mediterranean diet adherence (B = 0.51; β = 0.20; *p* = 0.008). Older age was also independently associated with higher MEDAS scores (B = 0.03; β = 0.15; *p* = 0.035). In addition, the olive-related preference score (B = 0.34; β = 0.16; *p* = 0.024) and olive-related consumption-frequency score (B = 0.42; β = 0.18; *p* = 0.014) were independently and positively associated with MEDAS score.

Sex, body mass index, smoking status, and physical activity were not significantly associated with MEDAS score (all *p* > 0.05).

Overall, these findings indicate that older age, greater preference for traditional dairy products, higher preference for olive-related items, and more frequent consumption of olive-related items were independently associated with greater Mediterranean diet adherence among the study participants.

To examine the potential influence of construct and measurement overlap between the olive-related independent variables and MEDAS, a sensitivity analysis was conducted using a modified MEDAS score excluding the two olive-oil-related components. In the sensitivity analysis (n = 265), neither the olive-related preference score (B = −0.25; β = −0.12; *p* = 0.146) nor the olive-related consumption-frequency score (B = 0.43; β = 0.11; *p* = 0.164) was significantly associated with the modified MEDAS score. Thus, the significant olive-related associations observed in the primary analysis were not retained after exclusion of the olive-oil-related MEDAS components, suggesting that the primary findings may have been influenced, at least in part, by construct and measurement overlap between the olive-related independent variables and the original MEDAS score. Complete results of the sensitivity analysis are presented in Supplementary Table S3.

## 4. Discussion

The present study investigated Mediterranean diet adherence together with preferences and consumption patterns for traditional dairy products and olive-related items among adults living in Cyprus. Using the study-specific descriptive categories, 40% of participants had MEDAS scores classified as good to excellent. Older age, greater preference for traditional dairy products, and greater preference for and more frequent consumption of olive-related items were independently associated with higher MEDAS scores [19,20,21,22]. Collectively, these findings indicate that preferences for traditional Mediterranean foods were positively associated with Mediterranean diet adherence within the study sample. However, given the cross-sectional design, the temporal direction of these associations cannot be established, and causal relationships cannot be inferred. Age was entered as a continuous variable in the multivariable model; therefore, the observed association with age was estimated across the age distribution rather than being based on a categorical comparison with the small subgroup of participants aged ≥ 60 years. Nevertheless, younger adults were disproportionately represented, while only 3.7% of participants were aged ≥ 60 years. Consequently, the observed association between age and Mediterranean diet adherence should be interpreted cautiously, particularly regarding its applicability to older adults.

Although the mean MEDAS score indicated moderate adherence, examination of individual MEDAS components revealed substantial variation in compliance with specific dietary recommendations. The use of olive oil as the principal culinary fat was almost universal among the study participants, consistent with its prominent role in traditional Cypriot dietary practices. In contrast, adherence to recommendations for fruit, vegetable, fish, and legume consumption was considerably lower, suggesting that some components of the traditional Mediterranean dietary pattern were more commonly followed than others within the study sample. This pattern is consistent with the broader nutrition transition documented across Mediterranean countries, where elements of traditional dietary practices increasingly coexist with more Westernised eating patterns [22,23,24].

Older participants demonstrated significantly greater adherence to the Mediterranean diet than younger adults. This finding is consistent with previous studies reporting higher Mediterranean diet adherence among older compared with younger adults [25,26]. Younger generations in Mediterranean populations have also been reported to show a gradual shift away from traditional dietary practices towards more Westernised dietary patterns, including greater reliance on highly processed and convenience foods [25,26]. Such generational changes in dietary habits represent an important public health concern, particularly in Mediterranean countries where the traditional dietary pattern forms part of the cultural and nutritional heritage. The lower adherence observed among younger participants in the present study highlights the importance of further investigating age-related differences in Mediterranean dietary practices.

In the present multivariable analysis, sex, BMI, smoking status, and physical activity were not independently associated with MEDAS score. These findings should be interpreted in the context of previous research, as associations between Mediterranean diet adherence and sociodemographic, anthropometric, and lifestyle characteristics have varied across populations [27]. The absence of statistically significant associations in the present analysis should not be interpreted as evidence that these factors are unrelated to Mediterranean diet adherence more generally, particularly given the demographic composition and convenience sampling of the present study. Preference for traditional dairy products was independently associated with higher MEDAS scores, indicating that participants with greater preference for traditional dairy foods also tended to report higher adherence to the Mediterranean diet. Traditional dairy products such as Halloumi, Anari and yoghurt remain culturally important foods in Cyprus and continue to contribute substantially to dietary habits. Beyond their nutritional value, these foods may also reflect stronger adherence to traditional eating practices and cultural food identity. Consequently, preference for traditional dairy products may serve as an indicator of broader dietary behaviours that are consistent with the Mediterranean dietary pattern. Similar observations have been reported in previous studies demonstrating that individuals who favor traditional foods generally exhibit healthier overall dietary patterns.

The present study also demonstrated a particularly strong preference for olive oil, which was both the most preferred and the most frequently consumed olive product. Approximately three-quarters of participants reported that they liked olive oil very much, while almost half consumed it daily. In contrast, although several other olive-derived products, including green olives, olive paste and olive oil bread spread, received generally favourable preference ratings, they were consumed considerably less frequently. The discrepancy between preference and consumption also indicates that liking a food does not necessarily translate into frequent consumption. Olive oil has a distinct culinary role as a staple ingredient used routinely in cooking, salad preparation, and food seasoning, which may facilitate its frequent consumption irrespective of specific meal occasions. In contrast, table olives, olive paste, and olive oil bread spreads may function primarily as complementary foods, condiments, or accompaniments and therefore be consumed less frequently despite favourable preference ratings. Other factors not assessed in the present study, including product availability, cost, convenience, household food practices, and perceptions of nutritional value or energy and salt content, could also influence the translation of preference into consumption. Future studies should directly assess these factors to clarify the observed preference–consumption gap and determine whether barriers to consuming traditional olive products exist. From a public health perspective, these findings suggest that interventions focusing solely on food liking may be insufficient; understanding practical barriers and the culinary contexts in which traditional foods are consumed may also be important.

Importantly, both preference for and consumption of olive-related items were independently associated with greater adherence to the Mediterranean diet after adjustment for demographic and lifestyle characteristics. These findings are biologically plausible because olive oil represents the principal source of dietary fat within the Mediterranean diet and is one of its defining characteristics. Olive oil is a defining component of the traditional Mediterranean dietary pattern, and its consumption contributes directly to several commonly used Mediterranean diet adherence indices, including MEDAS [2,10,12]. Nevertheless, these findings should be interpreted with some caution because olive oil consumption is itself included within the MEDAS score. Consequently, part of the observed association may reflect shared measurement between the olive-related independent variables and the MEDAS outcome rather than a completely independent relationship.

Sex, body mass index, smoking status, and physical activity were not independently associated with Mediterranean diet adherence after adjustment for the other variables included in the multivariable model. Similarly, no statistically significant sex differences were detected for the individual MEDAS components. These findings should not be interpreted as evidence of an absence of sex-related or other lifestyle differences in Mediterranean diet adherence more generally. In particular, women were substantially overrepresented in the present sample (70.7%), which limits the generalizability of sex-specific comparisons. Moreover, the relatively homogeneous demographic and educational characteristics of the study sample may have reduced variability in some characteristics and limited the ability to detect relatively small independent associations. Accordingly, the absence of statistically significant associations for sex, BMI, smoking status, and physical activity should be interpreted cautiously and within the context of the present study sample. The sensitivity analysis provided further insight into the potential construct and measurement overlap between the olive-related independent variables and MEDAS. After excluding the two olive-oil-related components from the MEDAS score, the associations of olive-related preference and consumption frequency with Mediterranean diet adherence were attenuated and were no longer statistically significant. This suggests that the significant associations observed in the primary analysis may be partly attributable to shared measurement between the olive-related independent variables and the original MEDAS score. Accordingly, the associations between olive-related behaviours and overall Mediterranean diet adherence should be interpreted cautiously.

The present study has several strengths. To our knowledge, this is the first study conducted in Cyprus to simultaneously examine Mediterranean diet adherence together with preferences and consumption patterns for traditional dairy products and olive-related items within the same analytical framework. The study included a relatively large sample of adults from different regions of Cyprus and incorporated validated assessment of Mediterranean diet adherence using the MEDAS questionnaire. Furthermore, the dairy preference questionnaire demonstrated excellent internal consistency and an interpretable component structure in the present sample. Multivariable regression analysis further enabled adjustment for important demographic and lifestyle characteristics when examining factors associated with Mediterranean diet adherence.

Several limitations should be acknowledged. First, the cross-sectional design precludes establishing temporality or causality between food preferences, consumption patterns, and Mediterranean diet adherence; therefore, the direction of the observed associations cannot be determined. Second, dietary intake, anthropometric measures, and lifestyle behaviours were self-reported and may be subject to recall and social desirability bias. In particular, self-reported height and weight may have resulted in some misclassification of BMI. Third, the online convenience sampling strategy, involving recruitment through social media, university mailing lists, professional networks, and community organisations, may have introduced substantial selection and self-selection bias, including preferential participation by individuals with greater interest in health or nutrition. The resulting sample was predominantly female (70.7%), relatively young (49.8% aged 18–29 years and only 3.7% aged ≥ 60 years), and highly educated (86.4% with undergraduate or postgraduate education). Given that these characteristics may be associated with dietary behaviours and Mediterranean diet adherence, the demographic imbalance substantially limits the external validity of the findings. Accordingly, neither the observed distribution of MEDAS scores nor the associations identified in this study should be interpreted as nationally representative estimates for the general adult population of Cyprus.

Fourth, the MEDAS score contains two olive-oil-related components, while olive-related preference and consumption-frequency scores were included as independent variables in the primary regression model, resulting in potential construct and measurement overlap. Importantly, in the sensitivity analysis excluding these two MEDAS components, the associations of olive-related preference and consumption frequency with the modified MEDAS score were attenuated and no longer statistically significant. This finding indicates that the olive-related associations observed in the primary analysis may have been influenced, at least in part, by shared measurement and should therefore be interpreted with particular caution. Fifth, although the food-preference questionnaire demonstrated excellent internal consistency and a satisfactory component structure in the present sample, the newly developed preference items have not yet undergone external validation in an independent population. Sixth, data collection extended over a 12-month period, and seasonal variation may have influenced the reported consumption of some foods. Finally, although the multivariable analysis accounted for several demographic, anthropometric, lifestyle, and food-related characteristics, socioeconomic variables, including education and household income, were not included in the primary regression model; therefore, residual confounding by socioeconomic position cannot be excluded. This consideration is particularly relevant given the limited variability in educational attainment within the study sample. Residual confounding by other factors, including nutrition knowledge, food availability, and cultural influences, is also possible.

Despite these limitations, the present study provides novel evidence that preferences for traditional dairy products and olive-related items are positively associated with Mediterranean diet adherence among adults in Cyprus. These findings identify culturally familiar food preferences as factors associated with Mediterranean diet adherence in this study sample. Longitudinal and intervention studies are needed to determine the direction of these relationships and whether changes in traditional food preferences are accompanied by changes in Mediterranean diet adherence.

## 5. Conclusions

In this convenience sample of adults living in Cyprus, greater preference for traditional dairy products and greater preference for and more frequent consumption of olive-related items were associated with higher Mediterranean diet adherence in the primary analysis. However, the olive-related associations were attenuated and no longer statistically significant in the sensitivity analysis after exclusion of the two olive-oil-related MEDAS components, indicating that these findings should be interpreted cautiously in view of potential construct and measurement overlap. More broadly, the observed associations between traditional food preferences and Mediterranean diet adherence are cross-sectional and sample-specific and therefore do not establish temporality or causality. Given the demographic characteristics and convenience sampling of the present study, the findings should not be considered representative of the general adult population of Cyprus. Longitudinal studies using more representative samples are needed to confirm these associations and establish their temporal direction, while intervention studies would be required to determine whether changes in traditional food preferences or consumption are accompanied by changes in Mediterranean diet adherence.

## Figures and Tables

**Table 1:** Sociodemographic, anthropometric and lifestyle characteristics of the study participants (N = 273).

Variable	Total (N = 273), n (%)
Sex	
Female	193 (70.7)
Male	80 (29.3)
Age group (years)	
18–29	136 (49.8)
30–44	81 (29.7)
45–59	46 (16.8)
≥60	10 (3.7)
Age, mean ± SD (years)	35.3 ± 11.6
Educational level	
Secondary education	37 (13.6)
Undergraduate degree	133 (48.7)
Postgraduate degree	103 (37.7)
BMI category	
Underweight	16 (5.9)
Normal weight	139 (50.9)
Overweight	89 (32.6)
Obese	29 (10.6)
Smoking status	
Non-smoker	212 (77.7)
Smoker	61 (22.3)
Occupational activity level	
Mainly sedentary	130 (47.6)
Walking several hours/day	92 (33.7)
Walking with physical effort	30 (11.0)
Heavy manual work	5 (1.8)
Other	16 (5.9)

Abbreviations: BMI, body mass index; SD, standard deviation.

**Table 2:** Mediterranean Diet Adherence Score (MEDAS) among the study participants (N = 273).

Variable	Value
Participants, n	273
Mean ± SD	8.1 ± 2.6
Median	8
Minimum	3
Maximum	14

MEDAS categories were defined for descriptive purposes in the present study and do not represent externally validated adherence thresholds.

**Table 3:** Mediterranean Diet adherence categories (N = 273).

Category	MEDAS Score	n (%)
Low	≤5	81 (29.7)
Moderate	6–7	83 (30.4)
Good	8	43 (15.8)
Very good	9	28 (10.3)
Excellent	≥10	38 (13.9)

MEDAS: Mediterranean Diet Adherence Screener. Total score ranges from 0 to 14, with higher scores indicating greater adherence to the Mediterranean diet. MEDAS categories were defined for descriptive purposes in the present study and do not represent externally validated adherence thresholds.

**Table 4:** Individual MEDAS item scores by gender and total sample (N = 273).

MEDAS Item	Total Yes n (%)	Total Mean ± SD	Men Yes n (%), (n = 80)	Women Yes n (%), (n = 193)	*p*-Value
Olive oil as main culinary fat	245 (89.7)	0.90 ± 0.30	69 (87.3)	176 (90.7)	0.539
≥4 tablespoons olive oil/day	49 (17.9)	0.18 ± 0.38	14 (17.7)	35 (18.0)	1.000
≥2 servings vegetables/day	67 (24.5)	0.25 ± 0.43	23 (29.1)	44 (22.7)	0.335
≥3 servings fruit/day	48 (17.6)	0.18 ± 0.38	14 (17.7)	34 (17.5)	1.000
<1 serving red meat/day	60 (22.0)	0.22 ± 0.41	17 (21.5)	43 (22.2)	1.000
<1 serving butter/margarine/cream/day	71 (26.0)	0.26 ± 0.44	25 (31.6)	46 (23.7)	0.229
<1 sugar-sweetened beverage/day	63 (23.1)	0.23 ± 0.42	21 (26.6)	42 (21.6)	0.472
≥3 glasses wine/week	21 (7.7)	0.08 ± 0.27	6 (7.6)	15 (7.7)	1.000
≥3 servings legumes/week	59 (21.6)	0.22 ± 0.41	21 (26.6)	38 (19.6)	0.266
≥3 servings fish/seafood/week	39 (14.3)	0.14 ± 0.35	15 (19.0)	24 (12.4)	0.220
<3 commercial sweets/week	75 (27.5)	0.27 ± 0.45	23 (29.1)	52 (26.8)	0.812
≥1 serving nuts/week	74 (27.1)	0.27 ± 0.44	22 (27.8)	52 (26.8)	0.979
Prefer poultry/rabbit over red/processed meat	95 (34.8)	0.35 ± 0.48	28 (35.4)	67 (34.5)	0.998
Sofrito-style dishes ≥2 times/week	91 (33.3)	0.33 ± 0.47	31 (39.2)	60 (30.9)	0.238

Abbreviations: MEDAS, Mediterranean Diet Adherence Screener. Each MEDAS item was scored as 1 = Yes and 0 = No. Values are presented as n (%) and mean ± SD. Sex differences were assessed using Pearson’s chi-square test. Sofrito refers to a traditional Mediterranean culinary preparation in which aromatic vegetables, typically onion and/or leek together with garlic and tomato, are slowly sautéed in olive oil to form a flavour base. It is commonly incorporated into dishes containing vegetables, legumes, rice, pasta, fish, or meat. In the MEDAS questionnaire, this item assesses consumption of dishes prepared with a tomato-, garlic-, onion-, or leek-based sauce made with olive oil. None of the individual MEDAS items differed significantly between men and women (all *p* > 0.05).

**Table 5:** Ten most preferred milk and dairy products among the study participants (N = 273).

Rank	Product	Participants Selecting “Like Very Much”, n (%)
1	Halloumi	201 (73.6)
2	Feta cheese	189 (69.2)
3	Strained yogurt (10%)	176 (64.5)
4	Anari	158 (57.9)
5	Whole milk (3%)	147 (53.8)
6	Reduced-fat milk (1.5%)	133 (48.7)
7	Goat milk (1.5%)	121 (44.3)
8	Strained yogurt Light (5%)	110 (40.3)
9	Kefalotyri	98 (35.9)
10	Strained yogurt (0%)	87 (31.9)

Footnote: Participants evaluated milk and dairy products using a five-point hedonic scale (1 = *Strongly dislike*, 2 = *Dislike*, 3 = *Neither like nor dislike*, 4 = *Like*, and 5 = *Like very much*). Products are ranked according to the percentage of participants selecting “Like very much”.

**Table 6:** Principal component structure of the dairy preference questionnaire following varimax rotation.

Component	Preference Domain	Rotated Sum of Squared Loadings	Variance Explained (%)	Cumulative Variance (%)
Component 1	Traditional cheeses	15.253	25.42	25.42
Component 2	Milk products	11.175	18.63	44.05
Component 3	Yogurt products	7.362	12.27	56.32
Component 4	Specialty dairy products	6.651	11.08	67.40

Principal component analysis was performed with varimax rotation. Four components with eigenvalues >1.0 were retained and together accounted for 67.4% of the total variance. Component labels were assigned according to the dairy products with the highest rotated component loadings. The complete rotated component loading matrix is provided in Supplementary Table S2.

**Table 7:** Most preferred olive-related items among the study participants (N = 273).

Rank	Product	Participants Selecting “Like Very Much”, n (%)
1	Olive oil	205 (75.1)
2	Green olives	132 (48.4)
3	Olive oil bread spread	115 (42.1)
4	Foods cooked with olive oil	109 (39.9)
5	Olive paste	105 (38.5)
6	Black olives	101 (37.0)

Overall olive-related preference composite score: 3.76 ± 1.15 (mean ± SD; possible range: 1–5). For the purposes of this study, “olive-related items” comprise olive oil; olive-derived products (green olives, black olives, and olive paste); and culinary uses of olive oil (olive oil used as a bread spread and foods prepared with olive oil). Participants evaluated the olive-related items using a five-point hedonic scale (1 = strongly dislike, 2 = dislike, 3 = neither like nor dislike, 4 = like, and 5 = like very much). Products are ranked according to the percentage of participants selecting “Like very much.” The overall olive-related preference composite score was calculated from the six preference items, with higher scores indicating greater overall preference.

**Table 8:** Frequency of consumption of olive-related itemsamong study participants (N = 273).

Product	Never, n (%)	Rarely, n (%)	1–2 times/week, n (%)	3–4 times/week, n (%)	Daily, n (%)
Green olives	57 (20.9)	128 (46.9)	64 (23.4)	21 (7.7)	3 (1.1)
Black olives	65 (23.8)	114 (41.8)	65 (23.8)	26 (9.5)	3 (1.1)
Olive oil	6 (2.2)	3 (1.1)	45 (16.5)	83 (30.4)	136 (49.8)
Olive paste	81 (29.7)	147 (53.8)	33 (12.1)	6 (2.2)	6 (2.2)
Olive oil bread spread	86 (31.5)	133 (48.7)	38 (13.9)	12 (4.4)	4 (1.5)
Foods cooked with olive oil	55 (20.1)	92 (33.7)	60 (22.0)	33 (12.1)	33 (12.1)

Overall olive-related consumption-frequency composite score: 2.53 ± 0.62 (mean ± SD; possible range: 1–5). Data are presented as number of participants (n) and percentage (%). Percentages were calculated using the 273 participants with complete consumption-frequency data. Consumption-frequency categories were coded as 1 = Never, 2 = Rarely, 3 = 1–2 times/week, 4 = 3–4 times/week, and 5 = Daily. The overall olive-related consumption-frequency composite score was calculated as the mean of the six item scores; higher scores indicate more frequent overall consumption of olive-related items.

**Table 9:** Multiple linear regression examining factors associated with MEDAS score.

Variable	B	SE	Standardized β	t	*p*-Value
Constant	3.71	0.83	—	4.47	<0.001
Age (years)	0.03	0.02	0.15	2.12	0.035
Sex	0.09	0.18	0.03	0.50	0.617
Body mass index (kg/m^2^)	−0.03	0.02	−0.07	−1.08	0.281
Smoking status	−0.16	0.24	−0.04	−0.67	0.504
Physical activity	0.12	0.10	0.06	1.20	0.231
Traditional dairy preference score	0.51	0.19	0.20	2.68	0.008
Olive-related preference score	0.34	0.15	0.16	2.27	0.024
Olive-related consumption score	0.42	0.17	0.18	2.47	0.014

Model statistics: R^2^ = 0.29; Adjusted R^2^ = 0.27; Standard error of the estimate = 1.86; F = 13.48; *p* < 0.001. Note: B, unstandardised regression coefficient; SE, standard error; β, standardised regression coefficient; MEDAS, Mediterranean Diet Adherence Screener. All independent variables were entered simultaneously into the model.

## Data Availability

The original contributions presented in the study are included in the article/Supplementary Material, further inquiries can be directed to the corresponding authors.

## Supplementary Materials

The following supporting information can be downloaded at https://www.mdpi.com/article/10.3390/foods15173024/s1: Supplementary Table S1. Description of traditional dairy products included in the preference questionnaire. Supplementary Table S2. Rotated component loadings of the dairy preference questionnaire. Supplementary Table S3. Sensitivity analysis of factors associated with the modified MEDAS score excluding the two olive-oil-related components.

## Abbreviations

The following abbreviations are used in this manuscript:

BMI	Body Mass Index
EEBKΕΠ	Cyprus National Bioethics Committee
KMO	Kaiser–Meyer–Olkin
MEDAS	Mediterranean Diet Adherence Screener
SD	Standard Deviation
SE	Standard Error
SPSS	Statistical Package for the Social Sciences
PDO	Protected Designation of Origin
