# Supplementary material for: Traditional Dairy and Olive Food Preferences Are Associated with Mediterranean Diet Adherence in Cyprus
Source: Foods. 2026 Aug 27;15(17):3024. doi: 10.3390/foods15173024 (PMC13565078; doi:10.3390/foods15173024)
Supplement: Supplementary file 1 [file foods-15-03024-s001.zip › foods-4473645-supplementary.pdf]

Supplementary Table S1. Description of traditional dairy products included in the preference questionnaire.

Product	Description
Halloumi	A traditional Cypriot semi-hard, brined cheese produced from sheep's and goat's milk, or mixtures with cow's milk. It has a high melting point and is commonly consumed fresh or grilled.
Anari	A traditional Cypriot whey cheese produced from the whey remaining after Halloumi manufacture. It is available in fresh and dried forms.
Feta cheese	A brined white cheese traditionally produced from sheep's milk or a mixture of sheep's and goat's milk.
Kefalotyri	A hard, salty cheese produced from sheep's, goat's, or mixed milk, commonly consumed grated or grilled.

Supplementary Table S2. Rotated component loadings of the dairy preference questionnaire

**Item**	Dairy preference item		Component 1	Component 2	Component 3	Component 4
**1**	Γάλα πλήρες 3%	Whole milk 3%	0.103	-0.061	0.435	-0.490
**2**	Γάλα ελαφρύ 1,5%	Semi-skimmed milk 1.5%	0.141	0.190	0.508	-0.387
**3**	Γάλα άπαχο 0%	Skimmed milk 0%	0.215	0.126	0.206	-0.551
**4**	Γάλα αιγινό 1,5%	Goat milk 1.5%	0.254	0.057	-0.051	-0.723
**5**	Γάλα εμπλουτισμένο με 40% ασβέστιο 1,5%	Calcium-enriched milk (40% calcium) 1.5%	0.246	0.112	0.180	-0.764
**6**	Γάλα χωρίς λακτόζη 1,5%	Lactose-free milk 1.5%	0.092	0.214	0.174	-0.604
**7**	Γάλα βιολογικό αιγινό 1,5%	Organic goat milk 1.5%	0.228	0.216	0.008	-0.746
**8**	Γάλα βιολογικό αγελαδινό 1,5%	Organic cow's milk 1.5%	0.314	0.124	0.172	-0.793
**9**	Γαλατάκι σοκολάτα	Chocolate-flavoured milk	0.198	0.036	0.439	-0.494
**10**	Γαλατάκι σοκολάτα χωρίς πρόσθετη ζάχαρη	Chocolate-flavoured milk with no added sugar	0.341	0.109	0.328	-0.534
**11**	Γαλατάκι μπανάνα	Banana-flavoured milk	0.324	0.134	0.222	-0.369
**12**	Στραγγάτο γιαούρτι 10%	Strained yogurt 10%	0.006	0.234	0.641	-0.183

**13**	Στραγγάτο γιαούρτι light 5%	Light strained yogurt 5%	0.039	0.251	0.629	-0.270
**14**	Στραγγάτο γιαούρτι 0%	Strained yogurt 0%	0.213	0.275	0.351	-0.285
**15**	Βιολογικό αιγινό γιαούρτι	Organic goat yogurt	0.262	0.378	0.044	-0.455
**16**	Γιαούρτι χωρίς λακτόζη	Lactose-free yogurt	0.394	0.289	0.181	-0.433
**17**	Στραγγάτο snack γιαούρτι 2% με μέλι	Strained snack yogurt 2% with honey	0.533	0.292	0.272	-0.165
**18**	Στραγγάτο snack γιαούρτι 2% με γκρανόλα και ξηρούς καρπούς	Strained snack yogurt 2% with granola and nuts	0.595	0.297	0.291	-0.164
**19**	Πρόβειο γιαούρτι	Sheep's milk yogurt	0.383	0.498	-0.110	-0.283
**20**	Στραγγάτο γιαούρτι κεράσι 0%	Strained cherry yogurt 0%	0.803	0.221	0.048	-0.140
**21**	Στραγγάτο γιαούρτι φράουλα 0%	Strained strawberry yogurt 0%	0.827	0.262	0.091	-0.066
**22**	Στραγγάτο γιαούρτι ροδάκινο 0%	Strained peach yogurt 0%	0.813	0.162	0.045	-0.070
**23**	Στραγγάτο γιαούρτι κράνμπερι, μύρτιλα, ηλιόσποροι, λιναρόσποροι 0%	Strained yogurt 0% with cranberries, blueberries, sunflower seeds, and flaxseeds	0.828	0.195	0.040	-0.070
**24**	Στραγγάτο γιαούρτι μπανάνα 0%	Strained banana yogurt 0%	0.844	0.138	0.076	-0.107
**25**	Γιαούρτι λειτουργικό βανίλια με φυτικές ίνες και προβιοτικά	Functional vanilla yogurt with dietary fibre and probiotics	0.847	0.159	0.090	-0.222
**26**	Γιαούρτι λειτουργικό ροδάκινο με φυτικές ίνες και προβιοτικά	Functional peach yogurt with dietary fibre and probiotics	0.880	0.172	0.050	-0.217
**27**	Γιαούρτι λειτουργικό φράουλα με φυτικές ίνες και προβιοτικά	Functional strawberry yogurt with dietary fibre and probiotics	0.867	0.195	0.057	-0.204
**28**	Γιαούρτι λειτουργικό κόκκινα φρούτα, γκρανόλα, φυτικές ίνες και προβιοτικά	Functional yogurt with red fruits, granola, dietary fibre, and probiotics	0.897	0.137	0.081	-0.181
**29**	Γιαούρτι λειτουργικό ολικής αλέσεως, μέλι, φυτικές ίνες και προβιοτικά	Functional yogurt with whole grains, honey, dietary fibre, and probiotics	0.850	0.165	0.093	-0.171

**30**	Γιαούρτι με φράουλα, δημητριακά, επικάλυψη σοκολάτα	Strawberry yogurt with cereals and chocolate coating	0.839	0.159	0.206	-0.067
**31**	Γιαούρτι με βανίλια, δημητριακά, επικάλυψη σοκολάτα	Vanilla yogurt with cereals and chocolate coating	0.840	0.114	0.221	-0.099
**32**	Γιαούρτι με μπανάνα, δημητριακά, επικάλυψη σοκολάτα	Banana yogurt with cereals and chocolate coating	0.813	0.100	0.205	-0.053
**33**	Φυτική κρέμα	Plant-based cream	0.399	0.028	0.551	-0.167
**34**	Κρέμα γάλακτος light 17%	Light dairy cream 17%	0.353	0.089	0.679	-0.225
**35**	Κρέμα γάλακτος 35%	Dairy cream 35%	0.261	0.071	0.670	-0.185
**36**	Sour cream	Sour cream	0.066	0.149	0.662	-0.174
**37**	Χαλλούμι	Halloumi	0.211	0.538	0.486	-0.098
**38**	Βιολογικό χαλλούμι	Organic Halloumi	0.338	0.513	0.269	-0.234
**39**	Αιγοπρόβειο χαλλούμι	Goat and sheep milk Halloumi	0.274	0.604	0.111	-0.177
**40**	Αιγινό χαλλούμι	Goat milk Halloumi	0.271	0.580	0.123	-0.162
**41**	Πρόβειο Χαλλούμι	Sheep milk Halloumi	0.254	0.678	0.159	-0.131
**42**	Κυπριακό τυρί/ χαλλούμι light	Cypriot cheese/Light Halloumi	0.203	0.595	0.332	-0.111
**43**	Αναρή	Anari	0.151	0.762	0.008	-0.155
**44**	Αναρή αλατισμένη	Salted Anari	0.190	0.804	0.001	-0.164
**45**	Αναρή χωρίς λακτόζη	Lactose-free Anari	0.344	0.568	-0.097	-0.266
**46**	Αναρή ξηρή	Dry Anari	0.270	0.740	-0.072	-0.169
**47**	Αναρή ξηρή χωριάτικη αιγιοπρόβεια	Traditional dry goat and sheep milk Anari	0.280	0.714	-0.197	-0.220
**48**	Αναρή ανάλατη	Unsalted Anari	0.341	0.714	-0.044	-0.164
**49**	Φέτα	Feta cheese	0.234	0.669	0.348	0.031
**50**	Κεφαλοτύρι	Kefalotyri cheese	0.227	0.596	0.383	0.021
**51**	Τυρί φέτες	Sliced cheese	0.235	0.622	0.474	0.074

**52**	Τυρί light	Light cheese	0.094	0.616	0.506	-0.006
**53**	Τρίμμα χαλούμι & αναρή	Grated Halloumi and Anari	0.057	0.638	0.469	0.020
**54**	Τρίμμα αναρή	Grated Anari	0.147	0.703	0.231	-0.029
**55**	Τυρί μοτσαρέλα τριμμένο	Grated mozzarella cheese	0.131	0.660	0.390	0.072
**56**	Μαργαρίνη	Margarine	0.392	0.123	0.534	0.106
**57**	Μαργαρίνη light	Light margarine	0.449	0.094	0.503	0.059
**58**	Μαργαρίνη vegan	Vegan margarine	0.427	0.009	0.431	-0.164
**59**	Αϊράνι	Ayran	0.351	0.096	0.014	-0.311
**60**	Κεφίρ 1,5%	Kefir 1.5%	0.369	0.123	-0.054	-0.244
**61**	other	Other	Excluded	Excluded	Excluded	Excluded

Note: Values are varimax-rotated component loadings from a four-component PCA of the 60 substantive dairy-preference items. The 61st field ('Other') contained no responses and was therefore excluded from PCA. Complete-case N = 156. Loadings are reported to three decimal places.
The 61-item dairy preference questionnaire included an "Other" option. Because no participants provided a response for this item, it was excluded from the component analysis. PCA was therefore performed on the remaining 60 dairy-preference items. The complete rotated component loading matrix for the 60 analyzed items is provided in Supplementary Table S2.

Supplementary Table S3. Sensitivity analysis of factors associated with the modified MEDAS score excluding the two olive-oil-related components.

Variable	B	SE	Standardized β	t	p-value
Age (years)	0.01	0.01	0.07	1.01	0.312
Sex	−0.16	0.35	−0.03	−0.46	0.648
Body mass index (kg/m²)	0.01	0.04	0.03	0.35	0.728
Smoking status	0.15	0.34	0.03	0.45	0.654
Physical activity	0.03	0.14	0.01	0.22	0.824
Traditional dairy preference score	0.06	0.16	0.03	0.40	0.691

Olive-related preference score	−0.25	0.17	−0.12		−1.46	0.146
Olive-related consumption-frequency score	0.43	0.31	0.11		1.40	0.164

Note: B, unstandardised regression coefficient; SE, standard error; β, standardised regression coefficient; MEDAS, Mediterranean Diet Adherence Screener. For the sensitivity analysis, a modified MEDAS score was calculated by excluding the two olive-oil-related components (use of olive oil as the principal culinary fat and consumption of ≥4 tablespoons of olive oil per day), resulting in a theoretical score range of 0–12. The regression model included the same independent variables as the primary analysis, entered simultaneously. The analysis included participants with complete data for the modified MEDAS score and all variables entered in the model (N = 265). The intercept is not displayed because the table focuses on the independent-variable estimates. Statistical significance was set at $p < 0.05$.
